# Beyond the boundaries of pigmentation and inflammation: understanding the mechanistic basis of melasma–rosacea comorbidity

**DOI:** 10.3389/fmed.2026.1821174

**Published:** 2026-05-11

**Authors:** Xueli Li, Huanyu Shi, Yanyan Feng

**Affiliations:** 1 Department of Dermatology, Sichuan University Affiliated Chengdu Second People’s Hospital, Chengdu Second People’s Hospital, West China School of Medicine, Sichuan University, Chengdu, China; 2 North Sichuan Medical College, Nanchong, China

**Keywords:** comorbidity, inflammation, mast cells, melanogenesis, melasma, rosacea, tranexamic acid

## Abstract

Melasma and rosacea are common chronic facial dermatoses traditionally regarded as discrete clinical entities. Melasma is primarily characterized by dysregulated melanogenesis and aberrant pigmentary control, whereas rosacea is defined by inflammatory dysregulation and vascular hyperreactivity. However, accumulating clinical observations and experimental evidence increasingly challenge this conventional separation, revealing substantial overlap in their epidemiologic profiles, triggering factors, histopathological features, and underlying molecular signaling pathways. In this review, we synthesize emerging data that support a unified pathogenic framework for melasma–rosacea comorbidity, conceptualizing these conditions not as isolated disorders but as interconnected phenotypic manifestations arising from shared pathobiological substrates. We particularly emphasize convergent mechanisms involving neurovascular dysregulation, inflammatory cascades, barrier impairment, and pigment–vascular crosstalk, which collectively may account for their frequent coexistence and overlapping clinical features. Meanwhile, we discuss the therapeutic implications of this comorbidity model, highlighting tranexamic acid and other agents with anti-inflammatory and pigment-modulating properties. In this review, we aim to systematically synthesize and critically evaluate current evidence on the association between melasma and rosacea, and to propose a unified pathogenic framework underlying their comorbidity.

## Introduction

Melasma is a common acquired facial hyperpigmentation disorder predominantly affecting women with Fitzpatrick skin types III–IV, traditionally attributed to ultraviolet exposure, hormonal fluctuations, and genetic susceptibility (1). In contrast, rosacea is classified as a chronic inflammatory dermatosis characterized by facial flushing, persistent erythema, papules, pustules, and telangiectasia (2), with immune dysregulation and neurovascular hyperreactivity as central pathogenic features (3). Based on these dominant clinical manifestations, the two conditions have long been regarded as distinct disease entities.

However, an increasing number of clinical observations have begun to challenge this traditional dichotomous framework. A cohort study demonstrated a notably strong association between melasma and rosacea, as well as atopic dermatitis (4). In addition, a subset of patients with melasma exhibit conspicuous background erythema and telangiectasia underlying hyperpigmented lesions (5). Such overlap suggests that, in these individuals, melanogenesis may occur within a persistently inflamed and immunologically active vascular microenvironment rather than representing a purely pigmentary process.

Epidemiologic patterns and clinical phenotypes further substantiate this perspective, demonstrating shared risk factors, anatomical predilection, and triggering stimuli between melasma and rosacea. Globally, the overall prevalence of rosacea is estimated at approximately 5.46%, with a clear female predominance. Traditionally, rosacea has been considered more prevalent among individuals with lighter skin phototypes, in whom prevalence rates may exceed 10% (6, 7). However, emerging data suggest substantial under recognition and underreporting in populations with darker skin tones. It is estimated that more than 40 million individuals with darker phototypes worldwide are affected by rosacea (8), with documented cases across Africa, Asia, and South America—regions characterized by a higher proportion of deeply pigmented skin—where prevalence rates may reach up to 10% (9). Melasma predominantly affects women of reproductive age (10), and is particularly prevalent among individuals with Fitzpatrick skin phototypes III–V, in whom reported prevalence ranges from 8.8 to 40% (11). Among Asian women of childbearing age, prevalence has been reported to be as high as 30% (12). Collectively, these data underscore pronounced sex- and ethnicity-related disparities in both conditions, suggesting shared susceptibility profiles shaped by hormonal, genetic, and environmental determinants.

**Figure 1:**
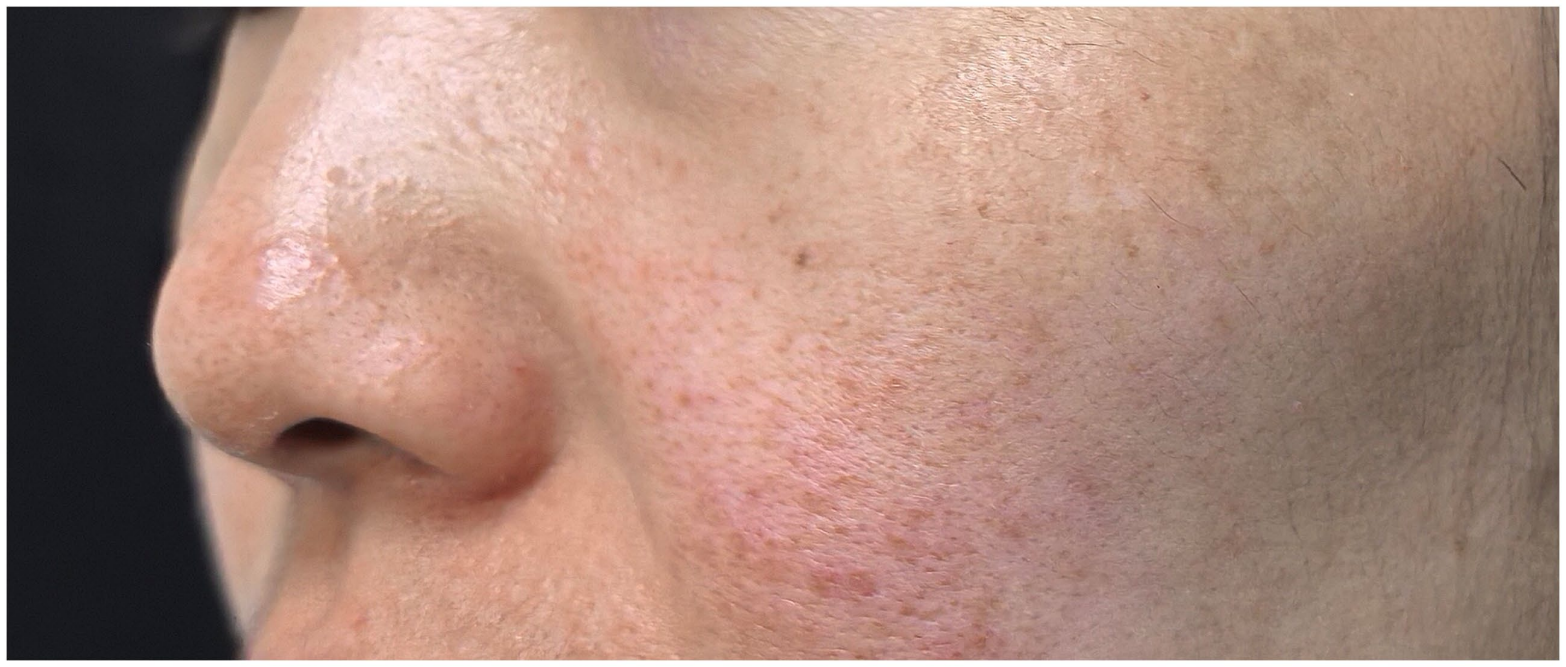
Representative clinical features of melasma–rosacea comorbidity. Representative clinical photograph of a patient presenting with overlapping features of melasma and rosacea. Central facial erythema, characteristic of rosacea, is observed on the cheeks, while hyperpigmented patches consistent with melasma are distributed over the malar regions. The coexistence of vascular and pigmentary changes highlights the clinical overlap between the two conditions. Written informed consent for publication of this image was obtained from the patient. The image has been anonymized to protect patient identity.

From an anatomical standpoint, both melasma and rosacea preferentially involve facial convexities, particularly the cheeks (The clinical features of patients with melasma–rosacea overlap are illustrated in Figure 1). Compared with concave facial regions, these areas are characterized by thinner epidermis, higher vascular density, and greater cumulative exposure to solar radiation, including ultraviolet (UV) and visible light spectra (13). Ultraviolet radiation not only stimulates melanocyte activation but also induces innate immune signaling, mast cell degranulation, angiogenesis, and extracellular matrix remodeling, thereby fostering a self-amplifying inflammatory microenvironment (14). Increasing evidence further indicates that visible light, particularly high-energy visible light (HEV), can induce persistent pigmentation and contributes to photo-induced skin damage through the generation of oxidative stress and inflammatory responses (15).

Importantly, failure to recognize the underlying inflammatory and immune-active background in melasma patients frequently leads to adverse outcomes. Energy-based or aggressive pigment-targeted therapies applied without addressing concomitant immune and vascular inflammation often trigger disease flares and post-inflammatory hyperpigmentation (16), underscoring the clinical relevance of an immunology-driven framework.

Therefore, rather than viewing melasma and rosacea as independent disorders, we propose that they represent interconnected manifestations along a shared cutaneous immune dysregulation spectrum. The objective of this review is to systematically synthesize and critically evaluate current epidemiological, mechanistic, and clinical evidence regarding their coexistence, and to develop a unified pathogenic framework integrating neurovascular, immune, and pigmentary pathways. This framework aims to inform more effective, mechanism-driven therapeutic strategies.

## Overlap of pathogenesis

### Mast cells as a central immunological hub linking melasma and rosacea

Mast cells are bone marrow–derived immune effector cells abundantly distributed within the dermis, particularly in close proximity to blood vessels, sensory nerve endings, and adnexal structures (17). Their cytoplasmic granules contain a broad repertoire of bioactive mediators, including histamine, heparin, proteases (tryptase and chymase), pro-inflammatory cytokines (IL-4, IL-6, TNF-*α*), and angiogenic and profibrotic growth factors such as vascular endothelial growth factor (VEGF), basic fibroblast growth factor (bFGF), and transforming growth factor-*β* (TGF-β). Through rapid degranulation and selective mediator release, mast cells play a pivotal role in orchestrating cutaneous inflammation, neurovascular responses, extracellular matrix remodeling, and pigmentary regulation (18).

In rosacea, microbial triggers and ultraviolet radiation stimulate keratinocytes to produce the antimicrobial peptide LL-37, a central mediator of innate immune activation. Beyond its antimicrobial function, LL-37 directly induces mast cell degranulation and potentiates downstream inflammatory signaling cascades, thereby amplifying local cutaneous inflammation (19). Moreover, LL-37 acts in concert with neuropeptides such as substance P (SP) and calcitonin gene–related peptide (CGRP) to activate mast cells via Mas-related G protein–coupled receptor X2 (MRGPRX2). This receptor has recently been recognized as a pivotal mediator of neuro–immune crosstalk in inflammatory dermatoses (20, 21).

Downstream of MRGPRX2, transient receptor potential vanilloid 4 (TRPV4) channels on mast cells are activated, leading to calcium influx and subsequent degranulation (22). Activated mast cells release histamine, proteases, cytokines, and angiogenic factors, collectively driving vasodilation, endothelial activation, increased vascular permeability, and leukocyte recruitment. These events culminate clinically in persistent erythema, telangiectasia, inflammatory papules and pustules, and neuropathic symptoms characteristic of rosacea (23).

Among mast cell–derived mediators, VEGF plays a central role in rosacea-associated vascular remodeling. Increased epidermal and dermal VEGF expression has been observed in areas of facial flushing, even at early disease stages, suggesting that angiogenesis is not merely a consequence but an integral component of rosacea pathogenesis (24). In addition, mast cell–derived tryptase activates protease-activated receptor-2 (PAR-2) on keratinocytes, inducing further release of SP and CGRP (25), thereby establishing a self-perpetuating neurogenic inflammatory loop (26). Emerging evidence also suggests dynamic crosstalk between mast cells and macrophages, neutrophils, and Th1/Th17 cells, although the precise contribution of these interactions to rosacea immunopathology requires further elucidation (23).

In melasma, mast cells are likewise markedly enriched within the lesional dermis (27), and contribute to disease progression through both direct and indirect mechanisms. Within the melasma microenvironment, ultraviolet radiation is considered as a principal exogenous trigger of mast cell activation. UV exposure not only directly induces mast cell degranulation but also indirectly enhances mast cell recruitment and activation via keratinocyte-derived mediators such as interleukin-1 (IL-1) and stem cell factor (SCF) (28, 29). Once activated, mast cells participate in pigmentary dysregulation by modulating vascular tone, extracellular matrix integrity, and melanocyte activity (30).

Histamine released from mast cells plays a pivotal role in melanogenesis (31). Experimental studies have demonstrated that histamine directly stimulates melanin synthesis through activation of the histamine H_2_ receptor on human melanocytes, thereby triggering the protein kinase A (PKA) signaling pathway and enhancing tyrosinase activity (32). Notably, this effect can be abrogated by H_2_ receptor antagonists but is not inhibited by H_1_ receptor blockade, indicating a receptor-specific mechanism underlying pigment regulation (33). Histamine-treated melanocytes exhibit dendrite elongation and increased melanin production, mechanistically linking mast cell activation to persistent hyperpigmentation in melasma.

**Figure 2:**
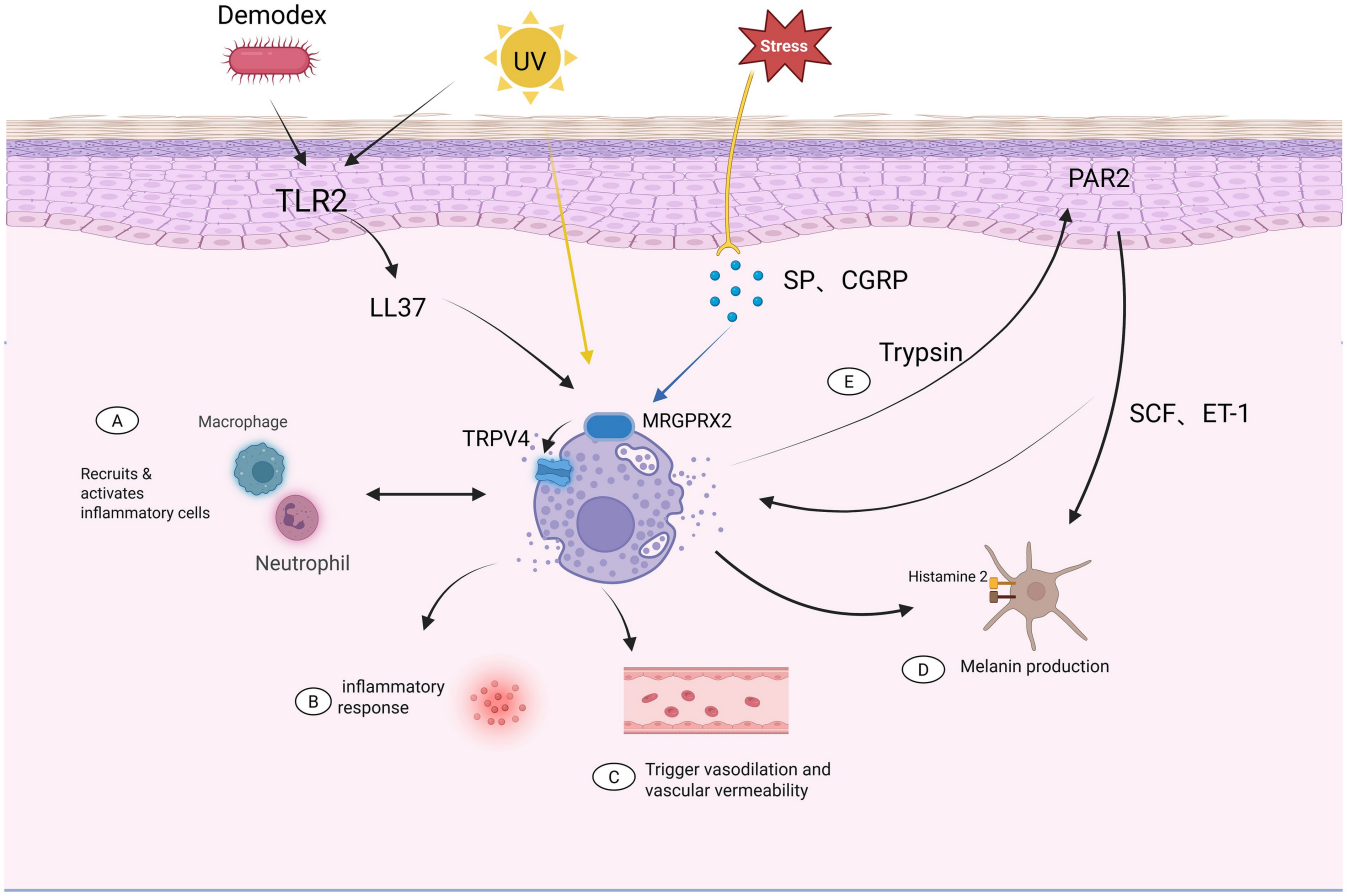
Mast cells function as a central immune hub linking the pathogenesis of melasma and rosacea. Environmental and endogenous triggers, including ultraviolet (UV) radiation, psychological stress, and microbial factors such as *Demodex*, initiate cutaneous responses through activation of keratinocyte Toll-like receptor 2 (TLR2), leading to increased expression of the antimicrobial peptide LL37. Concurrently, neurogenic stimuli promote the release of neuropeptides, including substance P (SP) and calcitonin gene-related peptide (CGRP), which activate mast cells via receptors such as MRGPRX2 and TRPV4. Activated mast cells function as a central integrative hub and mediate multiple downstream effects: (A) Recruitment and activation of inflammatory cells, including macrophages and neutrophils; (B) Amplification of the local inflammatory response through the release of cytokines and mediators; (C) Induction of vasodilation and increased vascular permeability, contributing to erythema and flushing; (D) Stimulation of melanocytes via histamine and related signaling pathways, promoting melanin production; (E) Crosstalk with keratinocyte-derived proteases (e.g., trypsin) and activation of protease-activated receptor 2 (PAR2), along with stem cell factor (SCF) and endothelin-1 (ET-1), further enhancing pigmentary and inflammatory signaling. Together, these interconnected pathways highlight the central role of mast cells in bridging neurogenic inflammation, vascular dysregulation, and melanogenesis, thereby contributing to the overlapping clinical manifestations of melasma and rosacea. Created with Biorender.com.

Beyond histamine, mast cells secrete VEGF, fibroblast growth factor-2 (FGF-2), and TGF-*β*, promoting angiogenesis and vascular dilation within melasma lesions (34). Clinically, increased dermal vessel density and enlarged vascular diameter correlate positively with pigmentation severity, reinforcing the concept that vascular alterations are functionally coupled to melanocyte activation (35). Moreover, mast cell–derived tryptase activates PAR-2 on keratinocytes, stimulating the release of melanogenic paracrine factors such as SCF and endothelin-1 (ET-1), which act on melanocyte c-Kit and endothelin receptor B, respectively, to enhance proliferation and melanin synthesis (36). The immunopathogenesis of melasma and rosacea is shown in Figure 2.

Taken together, mast cells may be regarded as a central immunological hub linking melasma and rosacea. By integrating neural signals, innate immune stimuli, and vascular responses, mast cells shape a dermal microenvironment characterized by chronic low-grade inflammation, angiogenesis, and enhanced melanogenic signaling. Although current evidence supporting the role of mast cells is largely derived from experimental studies and small-scale investigations, and direct clinical evidence establishing their causal contribution to melasma–rosacea comorbidity remains limited, we propose that this mast cell–centered shared pathway provides a mechanistic basis for the frequent clinical coexistence of these conditions and represents a compelling target for therapeutic intervention.

### Systemic remodeling of the dermal microenvironment

Chronic inflammation driven by mast cell activation profoundly reshapes the dermal extracellular matrix and basement membrane architecture. Ultraviolet radiation induces sustained upregulation of matrix metalloproteinases (MMP-2 and MMP-9), leading to degradation of type IV and VI collagen and disruption of basement membrane integrity (37); Mast cell–derived tryptase further amplifies this process by activating latent MMPs, accelerating collagen breakdown and dermal matrix destabilization (31).

Transcriptomic and immunohistochemical analyses reveal significantly elevated MMP-2 expression in melasma lesions compared with perilesional skin, implicating basement membrane loosening as a key pathogenic event (38, 39). Similarly, increased MMP-9 expression in rosacea skin reflects enhanced proteolytic activity associated with inflammation and tissue remodeling (40). Compromised barrier integrity facilitates penetration of ultraviolet radiation, microbial components, and chemical irritants, perpetuating innate immune activation and establishing a vicious cycle of chronic inflammation. Basement membrane disruption also enables bidirectional migration of inflammatory cells and pigmentary components. Melanin and melanocytes displaced into the superficial dermis are phagocytosed by macrophages, forming melanophages that contribute to pigment persistence and therapeutic resistance (27).

### Fine-tuned regulation of the neuro–immune–cutaneous axis

The neuro–immune–cutaneous (NIC) system is sustained by a highly dynamic and bidirectional communication network involving neuropeptides, cytokines, neurotransmitters, small-molecule mediators, and psychosocial stress–related signals (41). Through this integrated signaling circuitry, the skin functions not only as a physical barrier but also as a neuroendocrine and immunological interface capable of sensing and translating environmental stimuli into coordinated local and systemic responses. This system is essential for maintaining cutaneous homeostasis under physiological conditions, while its dysregulation predisposes to chronic inflammatory and pigmentary disorders (42).

Neurogenic factors play a pivotal role in the initiation and amplification of cutaneous inflammation. The peripheral nervous system is tightly interconnected with the central nervous system, endocrine pathways, and immune networks, forming a functional continuum rather than isolated compartments. In rosacea, external triggers such as heat, ultraviolet radiation, and psychological stress activate transient receptor potential (TRP) channels, particularly TRPV1, and TRPV4, expressed on cutaneous sensory nerve endings. Activation of these channels induces the release of neuropeptides, including substance P (SP), calcitonin gene-related peptide (CGRP), and vasoactive intestinal peptide (VIP), which collectively drive vasodilation, neurogenic inflammation, and heightened vascular reactivity (43, 44).

These neuropeptides exert pleiotropic effects on multiple skin-resident cell types. They directly act on vascular smooth muscle cells and endothelial cells to promote flushing and persistent erythema, while simultaneously stimulating mast cell degranulation and cytokine release, thereby amplifying inflammatory cascades (45). In parallel, keratinocytes, melanocytes, and immune cells express corresponding neuropeptide receptors, enabling neurogenic signals to influence barrier function, immune activation, and pigmentary regulation. The cutaneous neuroendocrine system, operating through local pathways analogous to the hypothalamic–pituitary–adrenal (HPA) axis, modulates stress and inflammatory responses (46); disruption of this regulatory loop contributes to the development of neurogenic rosacea (42). Sustained TRP channel hypersensitivity and continuous neuropeptide release not only underlie exaggerated vascular responses and burning sensations but also interact with emotional and psychological factors, resulting in refractory erythema and chronic pain syndromes.

**Figure 3:**
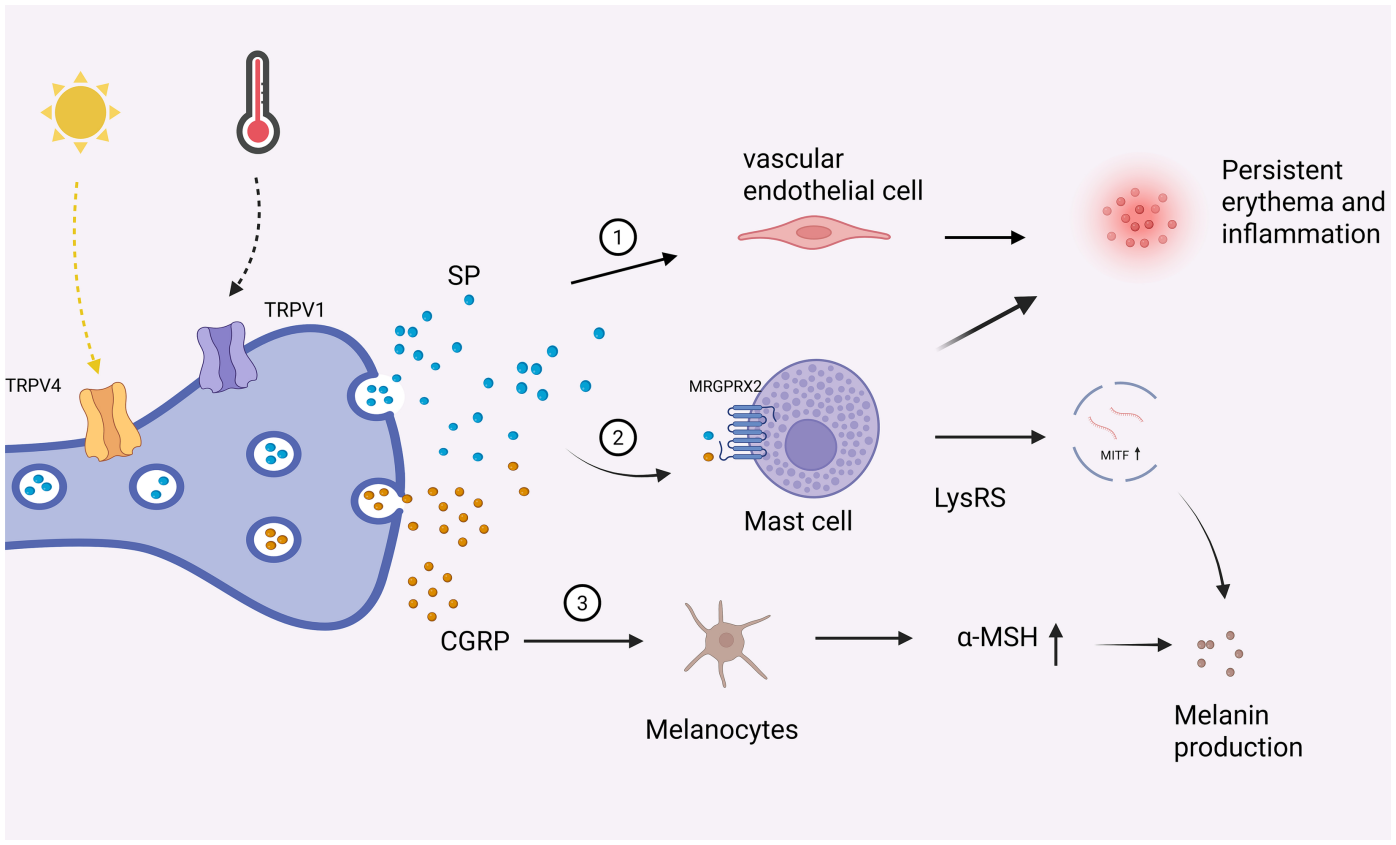
Neuro–immune mechanisms linking inflammation and melanogenesis in melasma–rosacea overlap. External triggers such as ultraviolet (UV) radiation and heat activate transient receptor potential (TRP) channels (TRPV1 and TRPV4) on sensory nerve endings, leading to the release of neuropeptides, including substance P (SP) and calcitonin gene–related peptide (CGRP). (1) SP and CGRP promote vascular endothelial activation, contributing to vasodilation and persistent erythema. (2) SP and CGRP also activate mast cells via MRGPRX2, inducing degranulation and the release of inflammatory mediators (e.g., LysRS), which upregulate microphthalmia-associated transcription factor (MITF) signaling and promote melanogenesis. (3) CGRP directly stimulates melanocytes and enhances α-MSH expression, further contributing to melanin production. These interconnected pathways highlight the central role of neurogenic inflammation and mast cell activation as key hubs linking vascular dysregulation and pigmentary changes, providing a mechanistic basis for the coexistence of rosacea and melasma. Created with Biorender.com.

Beyond their inflammatory and vascular effects, sensory neuropeptides directly participate in the regulation of melanogenesis. SP and CGRP released from sensory nerve endings act as key molecular bridges linking neurogenic inflammation to pigmentary alteration. SP activates mast cells via the Mas-related G protein–coupled receptor X2 (MRGPRX2), inducing nuclear translocation of lysyl-tRNA synthetase (LysRS) and subsequent upregulation of microphthalmia-associated transcription factor (MITF) activity (47), ultimately promoting melanin synthesis (48). In parallel, CGRP enhances melanocyte responsiveness to *α*-melanocyte–stimulating hormone (α-MSH), further amplifying melanogenic signaling (28). The neuropeptide-mediated signaling network is illustrated in Figure 3.

Collectively, these findings underscore the central role of sensory neuropeptides in coordinating inflammatory and pigmentary responses within the NIC axis. This integrated neuro–immune regulatory network provides a compelling molecular explanation for the frequent occurrence of melasma-like hyperpigmentation in patients with rosacea, and also suggests that neuromodulation may represent a promising therapeutic strategy, with the potential to simultaneously target vascular hyperreactivity, chronic inflammation, and aberrant melanogenesis. Despite these insights, current evidence is largely indirect, with most studies focusing on individual disease entities rather than their coexistence, and clinical validation of neuro–immune mechanisms in comorbid patients remains insufficient.

## Genetic and environmental susceptibility

Familial aggregation patterns support a contributory role of genetic predisposition in both melasma and rosacea. In melasma, disease susceptibility does not conform to a classical Mendelian inheritance model but instead reflects a complex polygenic architecture, wherein multiple genetic variants collectively shape baseline pigmentary regulation and modulate responsiveness to environmental and hormonal stimuli. Epidemiologic studies have demonstrated an increased prevalence among first-degree relatives, reinforcing the concept of an inherited susceptibility background. A Brazilian cohort reported that approximately 41–61% of affected individuals had at least one first-degree relative with melasma, including monozygotic twin sisters (49).

Genome-wide association studies (GWAS) and candidate gene analyses have identified several loci associated with melasma susceptibility, including melanocortin 1 receptor (MC1R), tyrosinase (TYR), H19, and peroxisome proliferator–activated receptor alpha (PPARA). Functionally, these genes converge on pathways governing melanocyte biology, oxidative stress responses, and hormone-sensitive metabolic signaling. The MC1R gene, located on chromosome 16q24.3, is highly polymorphic and exhibits marked ethnic variation (50). Notably, increased MC1R expression has been observed in the lesional epidermis of melasma, suggesting that enhanced melanocortin signaling may potentiate melanocyte responsiveness to ultraviolet and endocrine cues, thereby promoting persistent hyperpigmentation (51).

Rosacea likewise demonstrates heritable susceptibility, with approximately one-third of patients reporting a positive family history and a higher prevalence observed among individuals of Celtic and Northern European descent (52). A twin study involving 233 monozygotic and 42 dizygotic twin pairs confirmed a substantial genetic contribution to disease risk (53). Specific genetic variants, such as rs763035, as well as multiple HLA alleles, have been associated with rosacea and overlap with susceptibility loci implicated in autoimmune disorders, further underscoring a genetically mediated component of immune dysregulation (54, 55). More recently, large-scale GWAS have identified seven genome-wide significant loci linked to rosacea, some of which intersect with pigmentary biology. Variants within the HERC2–OCA2 region and SLC45A2, as well as an MC1R variant (rs1805007) approaching genome-wide significance, have been implicated in skin color determination and melanogenesis (56). These findings provide emerging genetic evidence connecting rosacea with pigment-regulatory pathways.

Environmental exposures further modulate this shared susceptibility landscape, with ultraviolet radiation representing the most prominent common trigger. In melasma, UV radiation directly stimulates melanocyte activity and disrupts dermal–epidermal homeostasis, resulting in excessive melanin production (57). Although rosacea is primarily characterized by vascular and inflammatory manifestations, UV exposure similarly exacerbates disease severity through the induction of oxidative stress, endothelial dysfunction, and innate immune activation (58). Facial vasculature in patients with rosacea exhibits heightened reactivity to physical and chemical stimuli—including heat, capsaicin, alcohol, and emotional stress—leading to exaggerated vasodilation (59). Notably, heat exposure and alcohol consumption have also been reported to aggravate hyperpigmentation in melasma.

Hormonal fluctuation represents another shared modulatory factor. Pregnancy and oral contraceptive use are well-established triggers for melasma (60), and have likewise been associated with an increased risk of rosacea (61), suggesting that endocrine variability may influence disease expression in both conditions. In addition, psychological stress can exacerbate disease activity through neuroendocrine pathways, forming a neuro–immune–cutaneous axis–mediated “stress–inflammation–pigmentation” self-amplifying loop (62, 63). A comparison of melasma and rosacea in terms of epidemiology and key pathogenic pathways is summarized in Table 1.

**Table 1:** Comparison of epidemiological features and pathogenic pathways between melasma and rosacea.

Category	Melasma	Rosacea
Comorbidity	OR = 5.1	
Genetic susceptibility	Familial aggregation; pigment-related genes (e.g. MITF regulation)	Approximately one-third of patients have a positive family history
UV radiation	Primary trigger of melanogenesis	Aggravates inflammation and vascular responses
Heat/stress	Indirect role via neuroendocrine signaling	Strong trigger via neurovascular dysregulation
Microbiota	Limited evidence	Strong association (e.g., Demodex, dysbiosis)
Neurogenic inflammation	Contributes to melanocyte activation	Central mechanism (flushing, erythema)
Mast cell activation	Promotes melanogenesis	Drives inflammation and angiogenesis
Vascular changes	Secondary role	Core feature (vasodilation, angiogenesis)
Melanogenesis pathway	Core pathogenic process (MITF)	Secondary or indirect

In concert, these genetic and environmental determinants delineate a shared susceptibility framework: inherited variants establish individual thresholds governing neurovascular, immune, and pigmentary responsiveness, whereas environmental exposures shape the ultimate phenotypic manifestation of disease.

## Implications for clinical management and therapeutic strategies

Recognition of melasma–rosacea comorbidity necessitates a fundamental shift in clinical management paradigms—from conventional single-target interventions toward mechanism-informed, multimodal therapeutic strategies. From a practical standpoint, in patients presenting with concurrent erythematous and pigmentary features, restoration of immune and neurovascular homeostasis should precede aggressive or energy-based pigment-targeted therapies. Interventions aimed at attenuating innate immune activation, stabilizing mast cell degranulation, and dampening neurogenic inflammation may serve as foundational treatments (3), thereby establishing a permissive cutaneous microenvironment for subsequent pigmentary or vascular modulation (64).

Photoprotection remains the cornerstone of management (65); however, within this conceptual framework, its significance extends beyond mere ultraviolet avoidance. Rather, it should be regarded as an immunomodulatory strategy designed to limit photoinduced neuroimmune activation. Similarly, therapeutic modalities that promote dermal remodeling and barrier restoration—such as energy-based devices applied under conditions of controlled inflammatory thresholds—may derive benefit not solely from direct lesion clearance but from interruption of chronic inflammatory feedback loops (66).

Pharmacologic interventions such as tranexamic acid (TXA) represent a paradigmatic example of mechanism-based therapy. Clinical studies have demonstrated that TXA exerts coordinated anti-inflammatory, antiangiogenic, and antimelanogenic effects, enabling simultaneous modulation of key pathogenic pathways involved in melasma–rosacea comorbidity (67). At the mechanistic level, TXA inhibits ultraviolet-induced plasmin activity in keratinocytes, thereby reducing the release of arachidonic acid and *α*-melanocyte–stimulating hormone (α-MSH), both of which serve as critical intermediaries linking photodamage to melanocyte activation (68). Concurrently, TXA downregulates Toll-like receptor 2 (TLR2) signaling, suppresses proinflammatory cytokine production—including IL-6 and TNF-α—and diminishes chemokine expression, thereby limiting innate immune activation. Notably, TXA also interferes with vascular endothelial growth factor (VEGF) signaling, limiting angiogenesis and vascular permeability, both of which are central features of rosacea and vascular-predominant melasma (64). Accumulating evidence further suggest that TXA modulates the local immune milieu by reducing mast cell proliferation and enhancing fibroblast activity, thereby contributing to extracellular matrix stabilization and barrier repair. This integrated mode of action may be particularly relevant in patients exhibiting concomitant inflammatory erythema and hyperpigmentation (69, 70).

Beyond tranexamic acid, other pharmacologic agents with both anti-inflammatory and pigment-modulating properties may also be considered within this shared mechanistic framework. Azelaic acid, a naturally occurring dicarboxylic acid, represents a prototypical example. It inhibits tyrosinase activity and mitochondrial redox pathways in melanocytes, while exerting anti-inflammatory effects through the suppression of reactive oxygen species and pro-inflammatory cytokines (71).

Clinically, azelaic acid has demonstrated efficacy in both melasma and rosacea (72), supporting its role as a potential “bridging therapy.” Monoclonal antibodies targeting neuropeptides have also shown efficacy in rosacea (73); however, their potential role in melasma remains to be established, and supporting evidence is currently limited.

Taken together, an integrated strategy grounded in the comorbidity framework—addressing inflammation control, vascular regulation, and pigment normalization in parallel—may achieve safer and more durable outcomes than phenotype-restricted interventions alone.

## Conclusion and perspectives

In summary, accumulating evidence suggests that melasma and rosacea, although traditionally regarded as distinct entities, share overlapping pathogenic mechanisms involving neurogenic inflammation, innate immune activation, mast cell signaling, vascular dysregulation, and melanogenesis. These interconnected pathways provide a unifying framework for understanding their frequent coexistence and clinical overlap.

Importantly, this integrated perspective has direct implications for clinical management. Conventional approaches that target isolated features, such as pigmentation or vascular abnormalities, may be insufficient to control disease activity in patients with comorbid presentations. Instead, mechanism-based, multimodal strategies aimed at restoring neuro–immune–vascular homeostasis may offer more effective and durable outcomes. Future research should focus on refining this integrative model and translating it into personalized therapeutic approaches for patients with overlapping phenotypes.

From a translational perspective, the recognition of melasma–rosacea comorbidity highlights the need to move beyond phenotype-based classification toward mechanism-driven stratification. Integrating clinical features with molecular and cellular signatures may enable more precise identification of patient subgroups and optimize therapeutic decision-making.

In this context, targeting shared upstream mechanisms, such as neurogenic inflammation and mast cell activation, represents a promising therapeutic direction. Furthermore, the development of combination strategies that simultaneously modulate inflammation, vascular function, and melanogenesis may improve treatment outcomes in patients with complex or overlapping disease presentations.

## Limitations and future directions

Despite the growing body of evidence supporting the comorbidity between melasma and rosacea, several important limitations should be acknowledged. First, much of the current evidence is derived from observational studies, small clinical cohorts, or experimental models, which limits the ability to establish causal relationships. Second, a substantial proportion of mechanistic insights—particularly those related to mast cell activation, neuro–immune interactions, and melanogenesis—are based on *in vitro* or animal studies, and their direct clinical relevance remains to be fully validated. Third, heterogeneity in study design, population characteristics, and diagnostic criteria across studies hampers direct comparison and synthesis of findings. In addition, many therapeutic strategies discussed are extrapolated from studies investigating melasma or rosacea independently, with limited data specifically addressing patients with confirmed comorbidity. Finally, genetic and environmental contributions are often inferred from population-based or self-reported data, which may introduce bias and limit generalizability. Collectively, these limitations highlight the need for well-designed, large-scale clinical studies and integrative translational research to better define the mechanistic links and optimize management strategies for melasma–rosacea comorbidity.

Future studies should incorporate multi-omics approaches, including transcriptomics and proteomics, to better characterize shared molecular signatures. In addition, clinical trials assessing the efficacy of tranexamic acid (TXA) and other emerging therapies in patients with comorbid presentations are warranted. The identification of reliable biomarkers, particularly those related to mast cell activation, may further facilitate patient stratification and targeted therapy. A deeper understanding of these mechanisms will be essential for advancing precision dermatology in pigmentary and inflammatory skin disorders.
