# Correction: Beyond the boundaries of pigmentation and inflammation: understanding the mechanistic basis of melasma–rosacea comorbidity

**DOI:** 10.3389/fmed.2026.1893292

**Published:** 2026-06-09

**Authors:** Xueli Li, Huanyu Shi, Yanyan Feng

**Affiliations:** 1 Department of Dermatology, Sichuan University Affiliated Chengdu Second People's Hospital, Chengdu Second People's Hospital, West China School of Medicine, Sichuan University, Chengdu, China; 2 North Sichuan Medical College, Nanchong, China

**Keywords:** comorbidity, inflammation, mast cells, melanogenesis, melasma, rosacea, tranexamic acid

In the published article, there was an error in the author affiliation. Affiliation 1 should be “Department of Dermatology, Sichuan University Affiliated Chengdu Second People's Hospital, Chengdu Second People's Hospital, West China School of Medicine, Sichuan University, Chengdu, China” instead of “Department of Dermatology, Chengdu Second People's Hospital, Chengdu, China.”

The original version of this article has been updated.

